# Case of a WHO Grade II Atypical Meningioma in a 16-Year-Old Female

**DOI:** 10.7759/cureus.37752

**Published:** 2023-04-18

**Authors:** Mahad A Khan, Haysum Khan, Bazeela Saeed, Inayat U Khan

**Affiliations:** 1 Medicine and Surgery, Islamabad Medical and Dental College, Islamabad, PAK; 2 Medicine and Surgery, Shifa Tameer-e-Millat University Shifa College of Medicine, Islamabad, PAK; 3 Neurology, Shifa International Hospital, Islamabad, PAK; 4 Neurological Surgery, Kulsum International Hospital, Islamabad, PAK

**Keywords:** brain tumors, cns tumors, who grade 2, atypical meningioma, benign tumors, parasagittal meningioma

## Abstract

Meningiomas have a high frequency of occurrence as primary intracranial tumors. We report the case of a 16-year-old female who presented with a three-week history of persistent headache, vomiting, and photophobia. Imaging studies revealed the presence of a meningioma in the right occipital lobe of the brain. The patient underwent surgical resection, and histopathological analysis confirmed the diagnosis of an atypical WHO grade 2 meningioma. The patient experienced a significant improvement in her symptoms postoperatively and had no evidence of recurrence on follow-up imaging. This case highlights the importance of considering meningioma in the differential diagnosis of relatively young patients presenting with chronic headaches, and the favorable prognosis associated with atypical WHO grade 2 meningiomas following complete surgical resection.

## Introduction

Meningioma is the most common central nervous system (CNS) tumor arising from the arachnoid cap cells associated with the dura mater or choroid plexus [1]. It accounts for 37.6% of all CNS tumors and 50% of all benign brain tumors, with an incidence of 37.75 per 100,000 in the 75-84 age group. It is more common in adults than in children. An incidence of only 0.14 per 100,000 is observed in children aged 0-19 years [2]. They have been classified into three grades according to the WHO, with grade 1 being slow-growing, grade 2 having an increased likelihood of recurrence, and grade 3 being malignant with metastatic potential. Factors that increase the risk of occurrence include family history, radiation exposure, and genetic disorders such as neurofibromatosis type 2 [3,4].

The clinical manifestations of meningiomas are primarily influenced by the location and size of the tumor, resulting in a diverse range of presentations. Hence, patients can be asymptomatic or exhibit signs of neurological deficits ranging from headaches, drowsiness, and seizures to visual disorders and even muscle weakness [3]. The gold standard for diagnosing meningioma remains brain magnetic resonance imaging (MRI), and at present the longstanding treatment strategies are mainly surgery and radiotherapy [3].

## Case presentation

A 16-year-old girl presented at the outpatient department with a persistent generalized headache that had been ongoing for three weeks. The headache had a sudden onset and was accompanied by symptoms such as nausea, vomiting, and photophobia. It had a throbbing sensation, was not associated with aura or seizures, and was particularly worse in the early morning. Although there were no aggravating factors, the headache was progressive in nature and partially relieved by intravenous (IV) paracetamol. Besides these complaints, the patient had no known underlying medical illness and no history of head trauma or falls. Additionally, the patient did not report any memory loss or personality changes.

The patient had also experienced six episodes of non-projectile and non-bilious vomiting over the past three weeks. There was no blood or mucus in the vomit, and it primarily consisted of food particles. The episodes mostly occurred in the morning, and the quantity of vomitus was half a cup. It was relieved by ondansetron injections. The patient did not report significant weight loss, and there were no signs of dehydration.

**Figure 1:**
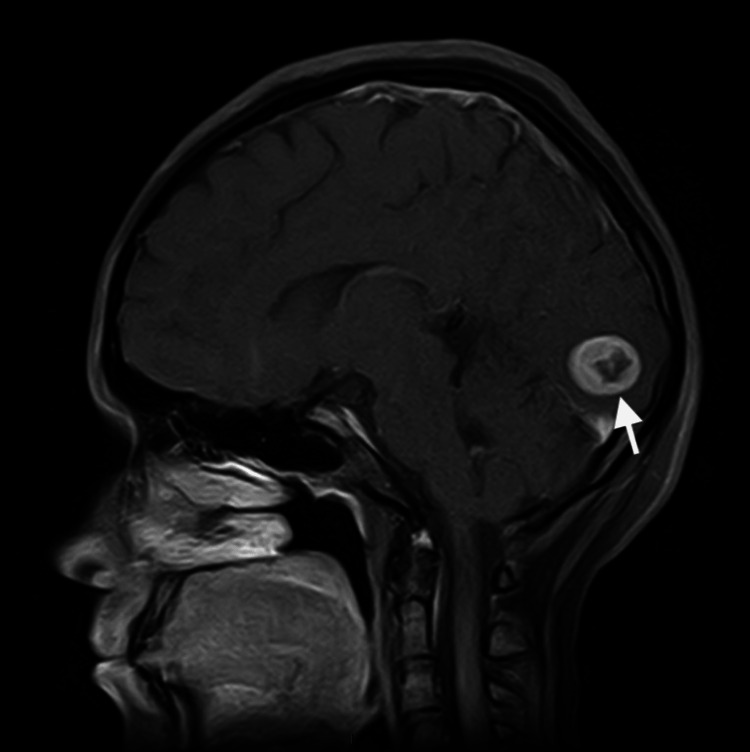
Preoperative magnetic resonance imaging scan in the T1 sagittal view. Arrow is pointing toward the meningioma.

**Figure 2:**
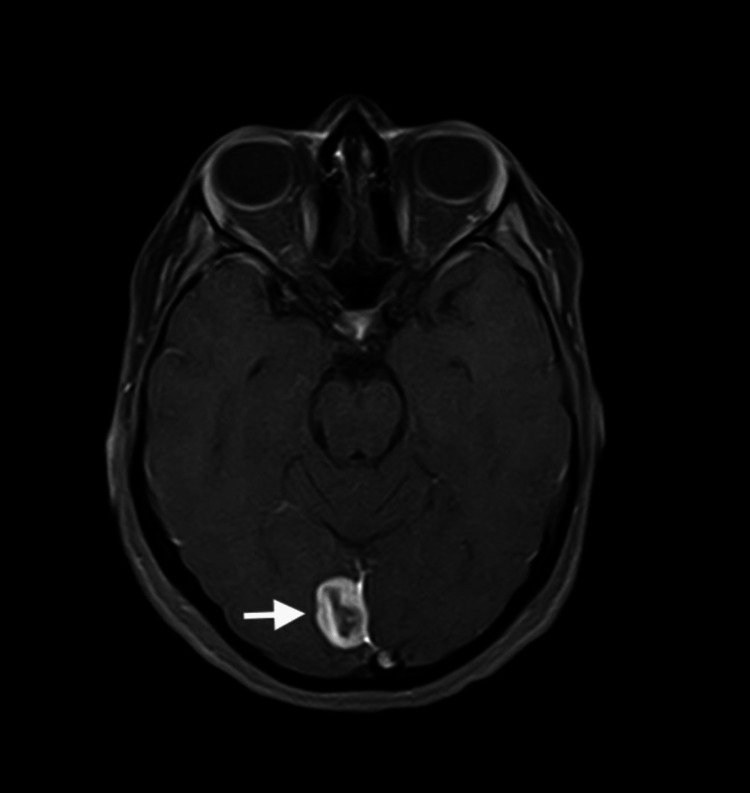
Preoperative magnetic resonance imaging scan in the T1 axial view. Arrow is pointing toward the lesion.

**Figure 3:**
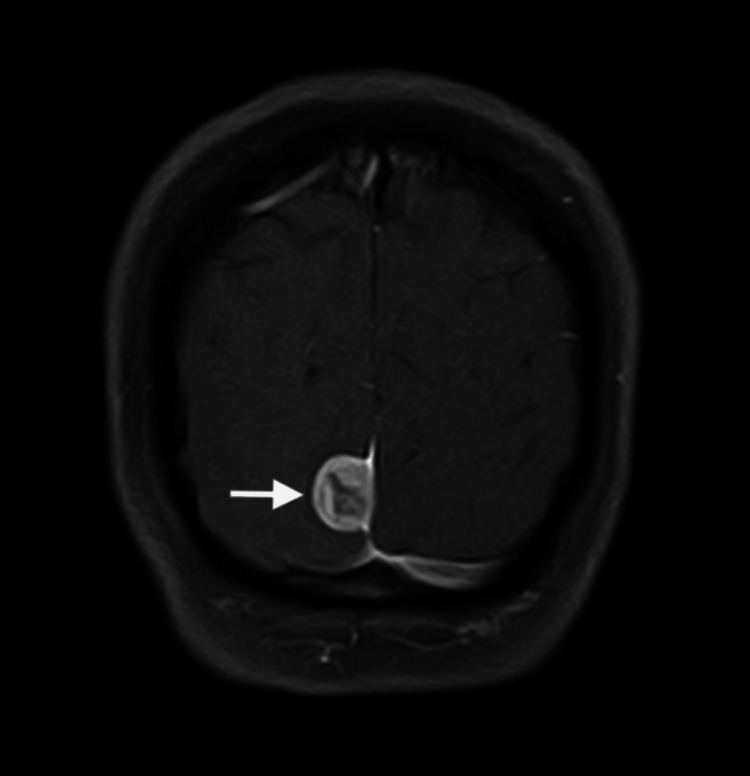
Preoperative magnetic resonance imaging scan at the T1 coronal view level. Arrow is pointing toward the lesion.

On presentation, the patient was alert, oriented, and cooperative. She was vitally stable and had a visual acuity of 6/6 in both eyes with no relative afferent pupillary defects or visual field defects. The patient had a Glasgow Coma Scale (GCS) score of 15/15 and did not have any motor defects. Apart from the complaints stated, neurological examination revealed no positive findings and a systemic review was also unremarkable. Blood tests performed as part of the routine investigations showed no hormonal imbalances, and she subsequently underwent a computed tomography (CT) scan without contrast, which revealed an ill-defined heterogenous lesion in the right occipital lobe parasagittal location. The MRI scan reported an approximately 15 × 21 mm extra-axial, intensely enhancing lesion along the right posterior interhemispheric fissure in the right parasagittal location at the level of the occipital lobe (Figures 1-3).

**Figure 4:**
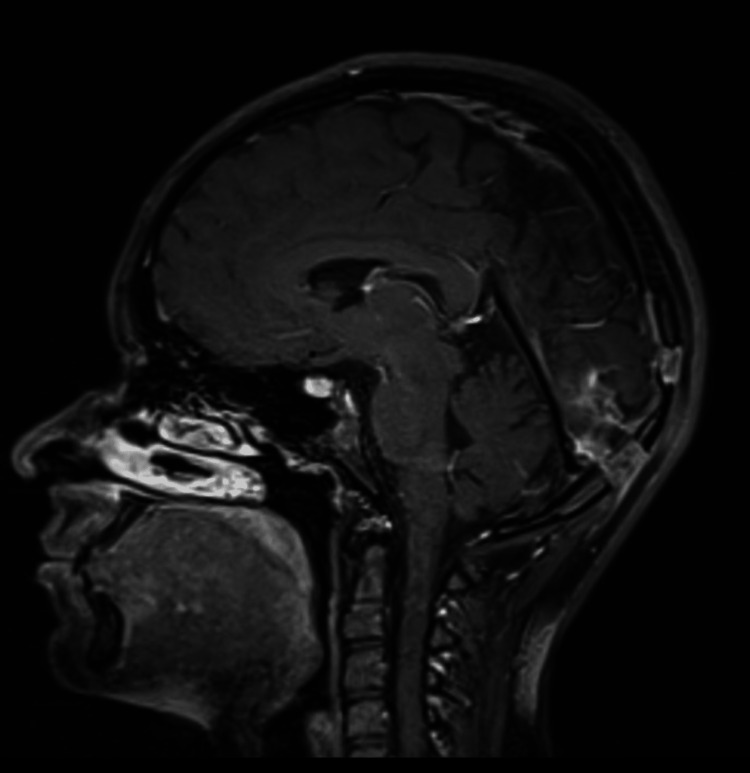
Postoperative magnetic resonance imaging scan in the T1 sagittal view. This scan was performed two months after the surgery and shows no visible enhancing lesion.

**Figure 5:**
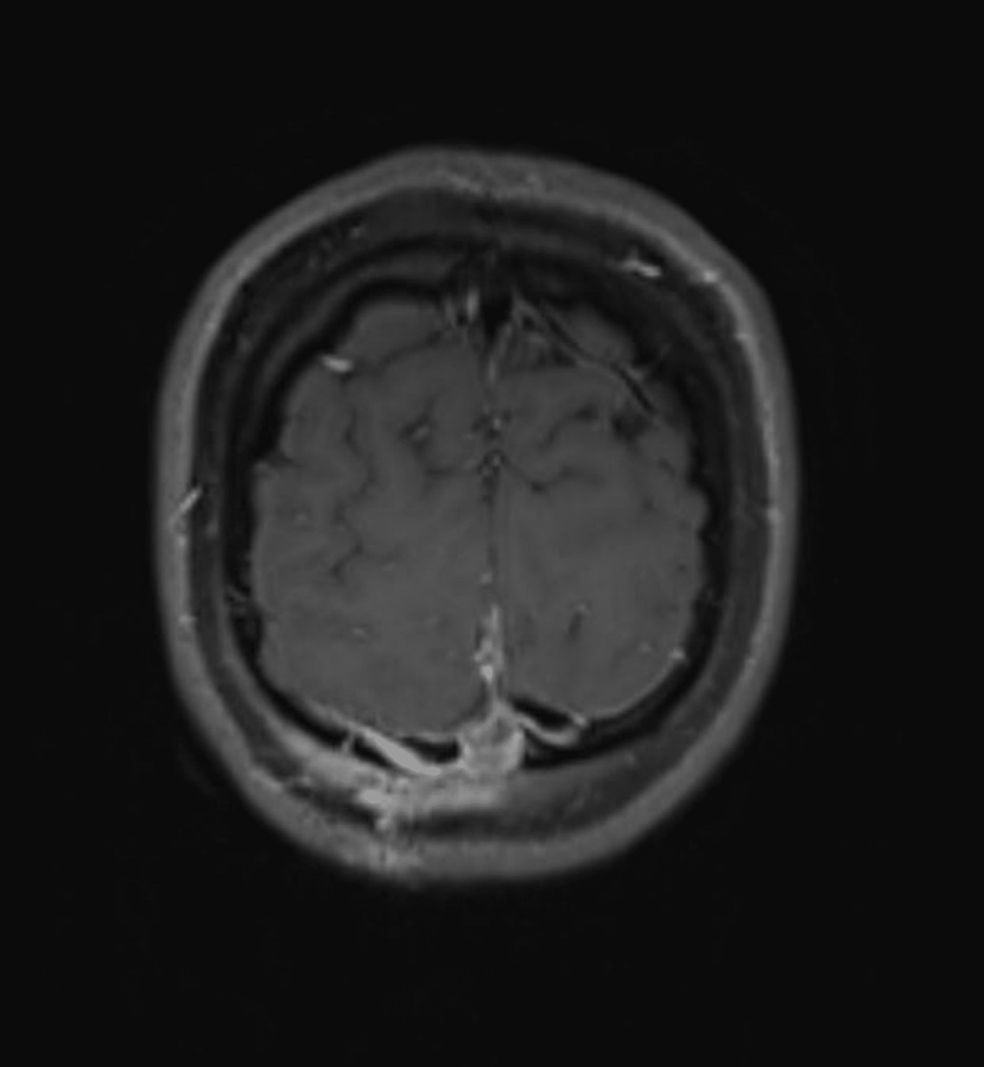
Postoperative magnetic resonance imaging scan in the coronal view. The scan does not show any residual disease.

After preoperative evaluation, the patient was placed under general anesthesia, and the neuronavigation system was calibrated. A craniotomy was then performed, followed by a dural opening. The meningioma was excised according to Simpson grade 1, along with coagulation of the region of the tentorium attached to it. Vessels supplying the tumor were coagulated and excised, and hemostasis of the brain was secured to ensure that there was no residual tumor or bleeding. Because the tumor had not invaded the dura or bone, they were left intact. Apart from mild anesthesia-related nausea and vomiting, the postoperative period was unremarkable. The patient was called for a follow-up visit two weeks after surgery to assess the wound and remove stitches, and then again after three months to undergo radiological investigations. MRI scan performed three months after surgery revealed reactionary subtle dural enhancement in the surgical bed, with no signs of residual disease. Additionally, there was no evidence of intracranial hemorrhage, established territorial infarct, or any abnormal enhancing lesion, and the patient showed marked improvement in her symptoms (Figures 4, 5).

## Discussion

As already established, meningiomas occur most commonly in the fifth decade of life, but they may rarely present in children having an incidence rate of 0.4-4.6% of all primary brain tumors in the age group of 0-18 years [5]. Moreover, according to the literature, tumors in children follow a more aggressive course than in their adult counterparts [6].

Meningiomas exhibit diverse symptoms, including headaches caused by elevated intracranial pressure, focal neurological impairments, and seizures. Lesions in the anterior or parasagittal location additionally present with personality changes and altered consciousness levels, which can be misdiagnosed as depression or dementia [7]. Primary complaints of our case were headaches along with nausea, vomiting, and photophobia.

**Figure 6:**
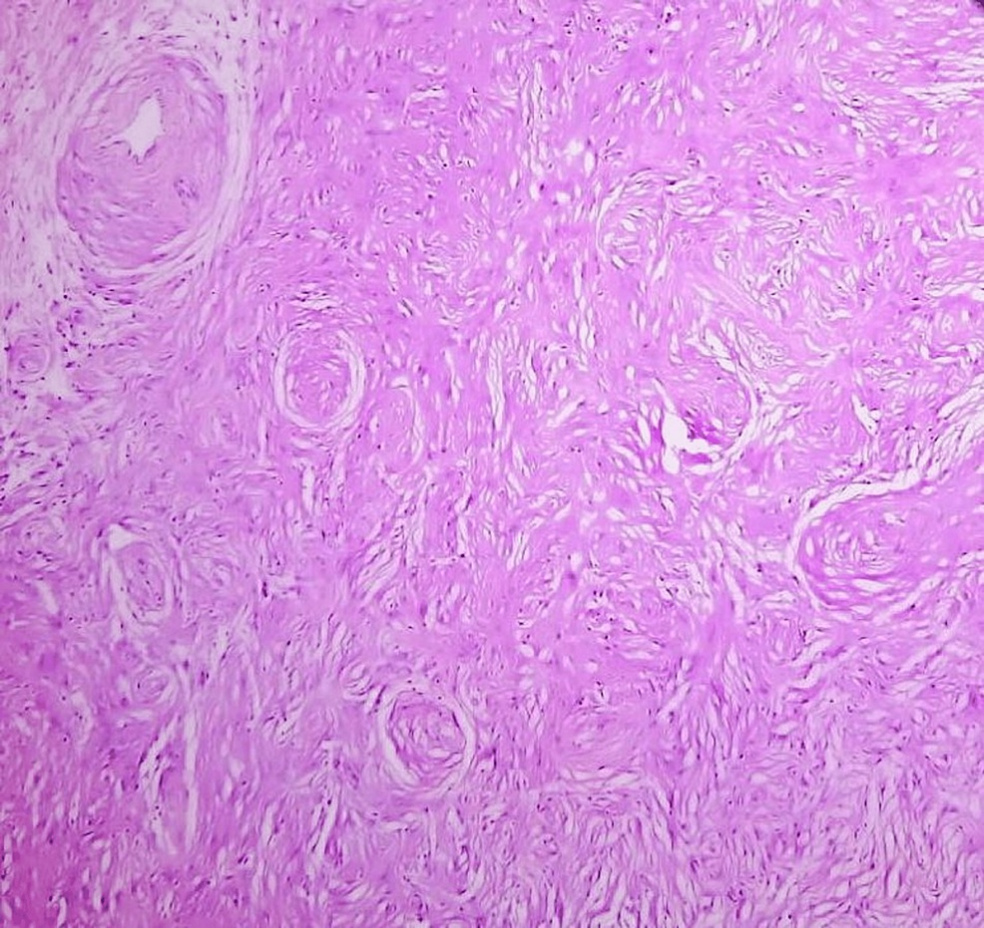
Menigothelial whorls (magnification ×10).

**Figure 7:**
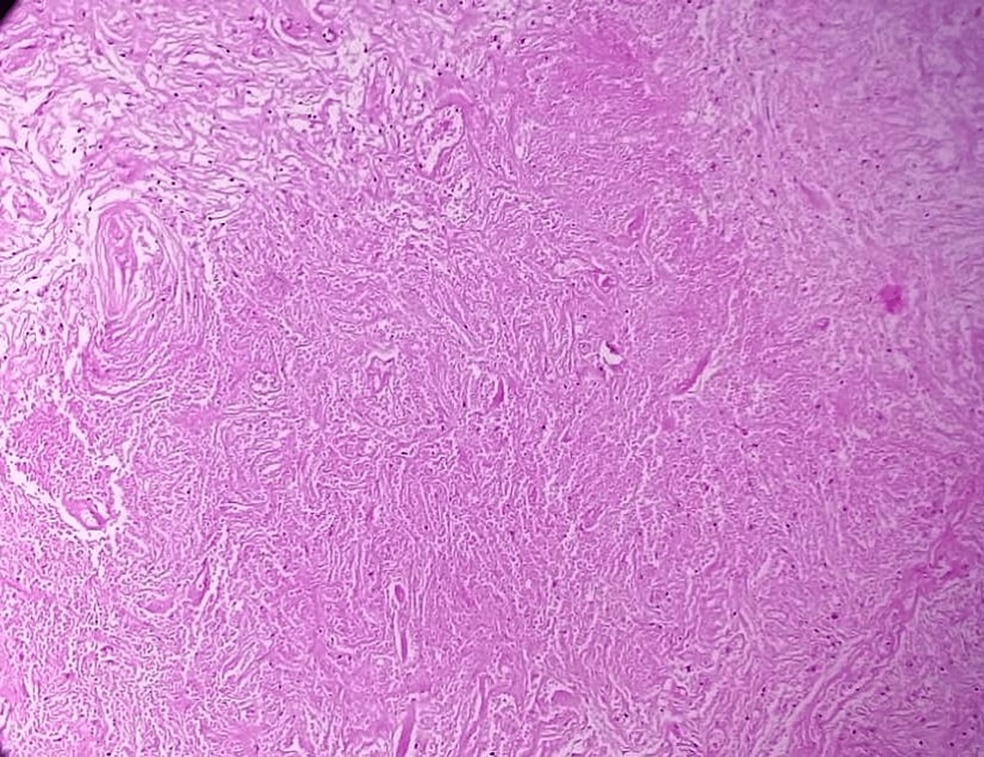
Foci of liquefactive necrosis (magnification ×10).

The histologic picture of a meningioma can vary depending on the specific type and grade of the tumor, but they generally have neoplastic cells arranged in a distinctive whorled pattern and psammoma bodies. The histologic images of the resected meningioma are depicted in Figure 6 and Figure 7.

The atypical (WHO grade II) meningioma variant possesses histologic and clinical characteristics that are intermediate between those of the benign and malignant types [8]. The recent WHO classification of tumors of the CNS classifies meningiomas as Grade 2 atypical if they fulfill the following criteria: if they demonstrate 4-19 mitotic figures per 10 high-power fields, invade the brain or show evidence of brain edema or necrosis, or show increased cellularity, nuclear atypia, and small foci of necrosis [8].

The mainstay treatment of meningiomas is surgery, but meningiomas with a diameter of less than 2.5 cm, without neurological symptoms, calcifications on brain CT scan, and hypo signal intensity on brain MRI can be observed with regular clinical and MRI follow-up [4]. To achieve long-term remission total excision should be done, but this is not always possible in the pediatric age groups, especially with large meningiomas, invasion of the surrounding structures, and brain parenchyma which leaves the option of radiotherapy which has proven to be very effective [9,10]. Meningiomas are further divided into grades, each with its specific treatment modality. Grade 1 meningiomas are usually treated with surgery or radiosurgery alone, and adjuvant radiotherapy is done only in cases of expanding remnants [11]. On the other hand, Grade 2 and 3 meningiomas are considered more aggressive and pose a greater risk of recurrence compared to Grade 1 tumors. Following gross total resection, these higher-grade meningiomas have been shown to have recurrence rates of up to 30-40% and 50-80% at five years, respectively, whereas Grade 1 tumors have a lower recurrence rate of approximately 10% [12-16]. Due to the high recurrence rates of Grade 2 and Grade 3 meningiomas, surgery along with adjuvant radiotherapy remains the mainstay of treatment [13,14,17].

A grading system known as Simpson introduced in 1957 is used to measure the recurrence rate depending on the extent of surgical resection. It consists of five grading systems, and it has been confirmed that for grade II meningiomas, for instance, Simpson 1 resection patients have a longer overall and progression-free survival [13].

## Conclusions

The literature suggests that surgical resection is the primary treatment for meningiomas, with adjuvant therapy being beneficial in cases where complete resection is not possible. In this case, advanced neuronavigation technology and microsurgical techniques were utilized by the surgeons to achieve successful resection of the meningioma while minimizing damage to the surrounding brain tissue. Radiological scans performed three months after surgery showed no signs of recurrence, and hence, adjuvant therapy was not administered. The patient showed marked improvement in her symptoms, and given the young age of presentation and successful surgical outcome with the use of neuronavigation, this case is worth presenting as a unique and informative case report.
